# Radon and thoron levels in the dwellings of Buddonithanda: a village in the environs of proposed uranium mining site, Nalgonda district, Telangana state, India

**DOI:** 10.1038/s41598-021-85698-1

**Published:** 2021-03-18

**Authors:** G. Suman, K. Vinay Kumar Reddy, M. Sreenath Reddy, Ch. Gopal Reddy, P. Yadagiri Reddy

**Affiliations:** 1 Department of Physics, Osmania University, Hyderabad, 500007 India; 2 Department of Physics, Chaitanya Bharathi Institute of Technology, Hyderabad, 500 075 India

**Keywords:** Environmental sciences, Natural hazards, Physics

## Abstract

Elevated levels of radon and thoron in the indoor atmosphere may cause the deleterious effects on the mankind. Mining sites and their environs attract a special interest in radon studies as higher levels are frequently reported in the habitats. In the present study, radon and thoron levels were measured in the dwellings of Buddonithanda, a village in the environs of proposed uranium mining site, with pin-hole (SSNTDs) dosimeters for the period of a year. The measured radon and thoron levels were found to vary widely from 14 to 675 Bq m^−3^ (geometric mean = 94 Bq m^−3^) and from 21 to 704 Bq m^−3^ (geometric mean = 121 Bq m^−3^), respectively. An attempt was made to understand the large spatial variation of these levels. The seasonal and diurnal variation studies were used in unraveling the behavior of the radioactive isotopes in indoor environment and the same was explained with the help of a simplified mathematical model. Quantification of inhalation dose due to radon and thoron was done with suitable occupancy factors.

## Introduction

Radon, being inert gas and radioactive in all of its isotopes, attracts much importance from radiological pollution point of view. The investigations across the globe indicate that half of the average annual natural background radiation dose comes from radon and its isotopes. Out of the various isotopes, the isotopes which are practically significant to the inhalation radiation dose are ^222^Rn and ^220^Rn, called as radon and thoron. The first one is more abundant, has half-life of 3.8 days and comes from the decay of ^226^Ra. The latter has a very short half-life of 55.6 s and comes from the decay of ^224^Ra^1^. Radon and thoron can enter human body by inhalation and most of the inhaled will be exhaled. However, a small fraction of the concentration of the gases might stuck in the lungs/ respiratory tract and these trapped radioactive elements on successive disintegration emit alpha particles that possibly ionize the lung tissues and results in the lung damage^2,3^. Epidemiological studies have confirmed that the exposure to radon in work places and dwellings increase the risk of developing lung cancer. Exposure to indoor radon and its isotopes have been determined to be the second leading cause of lung cancer after tobacco smoking^3^. The studies on uranium miners proved to be a positive risk coefficient on health and the measurements of radon and thoron levels are made inevitable in the indoor environment. This attention has been picked up the pace during last few decades and has become a global observable fact.

The concentration of radon and thoron gases in the indoors is largely influenced by the materials used for construction, life style of dwellers, geology and meteorological conditions of the study area^4^. Generally, the exposure to ^222^Rn and its daughter products contribute more to radiation dose than that of ^220^Rn^5^. However this perception has been changed, due to many systematic investigations across the world during the last few decades suggesting that, the ^220^Rn is also significantly contributes to the inhalation dose if the thorium content is rich in materials used for construction and local geology^6–8^.

Atomic Minerals Directorate for Exploration and Research (AMDER), Hyderabad, has conducted several geological studies in Chitrial area of Nalgonda district and established that it is one of the prospecting potential areas for uranium exploration^9,10^. The mining activity possibly enhances the environmental nuclear radiation levels in the adjoining areas. Buddonithanda, the village chosen for the present investigation, is situated in the close proximity of proposed mining site. This investigation aims to estimate radon, thoron levels and associated average annual effective inhalation dose to the population of Buddonithanda village. The pin-hole dosimeters were installed in the dwellings chosen randomly across the entire village covering different types of dwellings for the period of one year during November 2016 to October 2017 and covered nearly 45% of the dwellings of village.

The village Buddonithanda is located in the southern (south-east) part and almost 140 km away from the capital city (Hyderabad) of Telangana State. Most of the dwellings in the village were constructed with locally available materials such as bricks, stones, mud, dried gross, leaves and few are with cement and asbestos. This is the first systematic study in the neighboring village of proposed mining site and the outcomes of this investigation will be certainly a reference data for future studies to understand the effect of mining on the environmental nuclear radiation levels after mining comes into operation. The estimated radon and thoron levels in the different types of dwellings, seasonal/diurnal variation and inhalation dose due to radon and thoron to habitants of Buddonithanda village are discussed in this paper.

## Experimental techniques and methodology

Radon and thoron levels in the dwellings of Buddonithanda were measured with passive and active techniques namely, pin-hole dosimeters for time integrated measurements to account for seasonal variation and long term inhalation dose, RAD7 for short term measurements to account for diurnal variation.

### Pinhole based dosimeters

It is one of most versatile methods for mixed field environment of radon and thoron. It consists of two compartments separated by a central pinhole disc made up of High Density Poly Ethylene (HDPE) material. Each compartment has equal in dimensions of length 4.1 cm and radius 3.1 cm^11,12^. The mixture of radon and thoron gas from the ambient air enters dosimeter through a filter paper from face side and normally the filter paper obstructs the entry of unwanted particles from the environment. The ambient air containing radon and thoron gases enters the first compartment and radon gas diffuses through central pinhole disc (HDPE) to second compartment^13^. Hence the detector in the first compartment detects both radon and thoron while the second detects only radon because the diffusion of thoron to this compartment is impractical due its short half-life.

### RAD7 for simultaneous measurements

The RAD7 is a versatile comprehensive device used to assess the radon and thoron concentration instantaneously in the indoors and outdoors from the ambient air. It is solid-state semiconductor detector-based device in which the detection limit of radon and thoron is in between 4 and 400,000 Bq m^−3^. Since RAD7 is a humidity-sensitive device, KMnO_4_ is used as a desiccant^14,15^. In the present investigation, the diurnal variation of indoor radon and thoron concentration was studied using RAD7 [manufactured by DURRIDGE Company, USA. https://durridge.com/products/rad7-radon-detector]. The measurements were carried out in a dwelling of the study area for the complete cycle of a day with one hour interval.

### Experimental techniques

Measurements were carried out in the dwellings of Buddonithanda village using pinhole dosimeters with Solid State Nuclear Track Detector (SSNTD) and these are LR-115 type-II, strippable films. These SSNTDs were placed at specific position in the dosimeter; dosimeters were installed in dwellings approximately 1ft below from the ceiling and a minimum of 10 to 15 cm distance from the adjoining walls. The dosimeters were left in the dwelling for about three months, after the stipulated period the detectors were retrieved and reinstallation was done with a fresh batch of detectors. This process is repeated for a period of a year on quarterly basis and retrieval rate in the present study is nearly 73%. The retrieved SSNTDs were chemically etched using 2.5 N of NaOH solution at 60 °C for 90 min in constant temperature bath without stirring. The etched films were dried at room temperature and the recorded tracks were counted using a spark counter. The track densities were estimated into activity concentration using the calibration factors 0.017and 0.010 tr cm^−2^ per Bq d m^−3^ for radon and thoron^11,16^ respectively.

Annual effective inhalation dose due to radon and thoron in (mSv y^−1^) was calculated for indoors with the below formula by assuming the dose conversion factor for radon as 9 nSv y^−1^ and for thoron 40 nSv y^−1^^1^ and with appropriate occupancy factor.

1 $$Dose\;due \;to \; radon = C_{{{\text{Rn}}}} \left( {{\text{Bq}}\,{\text{m}}^{ - 3} } \right)*0.40*8760\,{\text{h}}*OF*9nSv\left( {{\text{Bq}}\,{\text{h}}\,{\text{m}}^{ - 3} } \right)^{ - 1} *10^{ - 6}$$

2 $$Dose\;due\;to \; thoron = C_{{{\text{Tn}}}} \left( {{\text{Bq}}\,{\text{m}}^{ - 3} } \right)*0.12*8760\,{\text{h}}*OF*40nSv\left( {{\text{Bq}}\,{\text{h}}\,{\text{m}}^{ - 3} } \right)^{ - 1} *10^{ - 6}$$

where C_Rn_ and C_Tn_ are the concentration of radon and thoron; *OF* is occupancy factor; the factors 0.40 and 0.12 are taken as equilibrium factors for radon and thoron^1,12^ respectively.

## Results and discussions

### Radon and thoron levels

The annual average concentrations of radon and thoron in the dwellings of Buddonithanda village measured during November 2016 to October 2017 are shown in Table 1. The estimated activity concentration is found to be varied for radon from14 to 675 Bq m^−3^ with geometric mean of 94 Bq m^−3^ (average value is found to be 122 ± 98 Bq m^−3^) and for thoron from 21 to 704 Bq m^−3^ with geometric mean of 121 Bq m^−3^ (average value is observed to be 166 ± 159 Bq m^−3^). The measured average concentration of radon and thoron levels is comparatively higher than the global average of 40 Bqm^−3^ for radon and 10 Bqm^−3^ for thoron^1^. It can be observed that, the radon and thoron levels in the study area are largely varied and distributed arbitrarily in a small region. The wide fluctuation in airborne radioactivity levels in the small area is unexpected and this may be attributed to the different category dwellings and varied life style of the residents though there is no observable difference in geology within the study area.

**Table 1:** The average concentration of radon and thoron (Bq m^−3^).

S. no	No. of measurements	Radon (^222^Rn)	Thoron (Rn^220^)
BT01	3	104 ± 58	93 ± 35
BT02	4	147 ± 74	219 ± 197
BT03	1	136	28
BT04	4	125 ± 69	271 ± 219
BT05	2	98 ± 56	267 ± 244
BT06	4	120 ± 77	147 ± 21
BT07	4	150 ± 117	216 ± 114
BT08	4	83 ± 31	101 ± 47
BT09	3	62 ± 19	135 ± 79
BT10	3	106 ± 79	104 ± 46
BT11	1	14	172
BT12	4	69 ± 49	179 ± 132
BT13	3	196 ± 132	109 ± 77
BT14	2	187 ± 117	218 ± 216
BT15	3	107 ± 62	149 ± 115
BT16	2	395 ± 395	373 ± 190
BT17	4	93 ± 17	226 ± 101
BT18	4	131 ± 63	247 ± 235
BT19	2	108 ± 1	43 ± 12
BT20	4	125 ± 76	104 ± 50
BT21	3	68 ± 37	112 ± 14
BT22	3	66 ± 9	141 ± 35
Min	–	14	21
Max	–	675	704
Average	–	122 ± 98	166 ± 159
GM	–	94	121

### Statistical distribution of ^222^Rn and ^220^Rn activity concentrations

**Figure 1:**
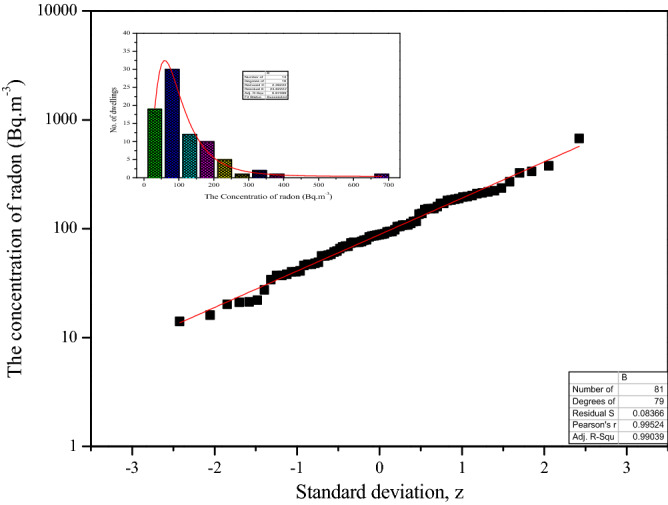
Distribution of the activity concentration of ^222^Rn. Histogram (inset).

**Figure 2:**
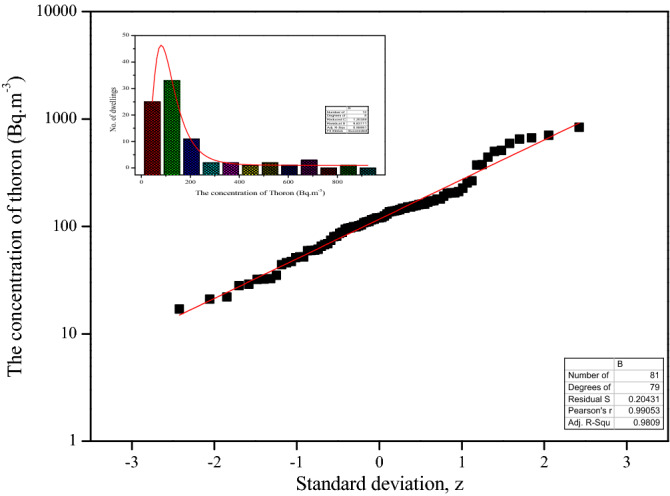
Distribution of the activity concentration of ^220^Rn. Histogram (inset).

To understand the wide range of the activity concentrations of radioactive isotopes, the best and yet simple statistical tools, histogram and probability distribution plots are employed. The distribution of measured radon and thoron levels in the study area for a period of one year for all the measurements is shown in Figs. 1 and 2, respectively. The insets depicted in the respective figures are histograms of the activity concentrations. The results show that about 16% and 11% of dwellings were recorded the below the 40 Bq m^−3^, about 30% and 37% of dwelling were recorded the average radon and thoron levels above 150 Bq m^−3^, respectively. It is observed that the radon and thoron activity followed lognormal distribution. It is very familiar that the radon/thoron activity concentrations follow log-normally in large areas^17–19^ but it is unexpected that the radon levels varying widely in a small area^12^. The asymmetric distribution of radon and thoron concentrations around the average or mean indicates the disproportionate weight of higher concentrations over the lower ones in the computation of the arithmetic mean. This problem can be avoided by taking the geometric mean and, thus, this statistics is the one recommended generally by radon and thoron experts for estimating the average values of concentration^17^. The maximum (max) concentration is based on the few farthest values in the distribution and the minimum (min) concentration is usually reported by large numbers of measurements^19^. The spatial variation of radon and thoron levels in the present study area is consistent with the earlier findings^18^, although they are not confined to a small region. This indicates that the influential parameters of residential radon like (1) *the life style of the people* (2) *structure of dwellings* may not be the same for each case despite the fact that the geology is same.

### Diurnal variation

**Figure 3:**
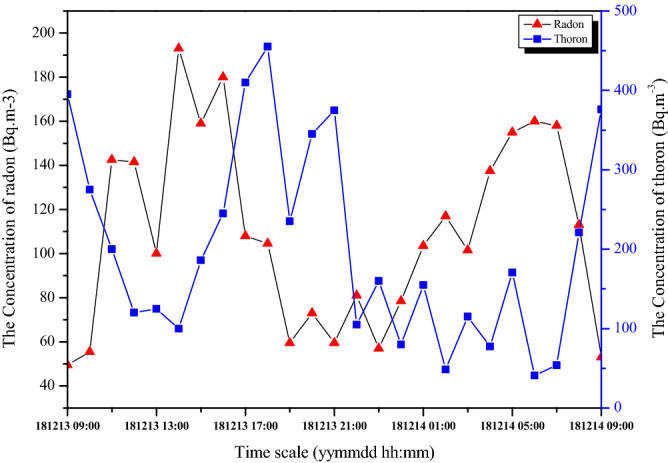
Diurnal variation of indoor radon and thoron.

The diurnal behaviour of indoor radon and thoron levels helps in understanding the variation of these levels with life style of dwellers. The simultaneous measurements were made with RAD7 in a selected dwelling in winter season. The acquisition of the data started at 9:00 am on February 01, 2018 and continued till next day morning in the intervals of one hour. The measured radon and thoron levels are shown in Fig. 3. The concentration of radon is showing steadily growing trend till around 17:00 h, since the dwellers are generally move to fields by closing the dwellings and as a result the radon gas accumulation is taking place. The radon levels show a dwindling trend in the sundown time because human activity increases in the dusk. Usually when the dwellers move to the dwellings, it may enhance the air circulation. During the night time the radon levels are raised to a maximum again as the ventilation is poor. The activity concentration of the gas decreases to a minimum in the morning time as the human activity begins. The increase in air exchange rate and the decrease in temperature gradient cause the low activity. The similar observations were also reported by Xie et al.^20^ and Suman et al.^12^.

Several researchers studied indoor radon levels with mathematical models^21,22^. Most of the mathematical models are based on following mass balance equation

3 $$Indoor\;radon\;concentration = Radon\;entry \pm Radon\;exchange - Decay$$

The rate change of indoor radon concentration, treating all sources contributing the indoor activity concentration constant, is given by the following equation^23^.

4 $$\frac{{dC_{{{\text{Rn}}}} }}{dt} = E_{{{\text{Rn}}}} + vC_{{{\text{Ro}}}} - \left( {\lambda_{{{\text{Rn}}}} + v} \right)C_{{{\text{Rn}}}}$$

where *C*_Rn_ is indoor radon concentration; *C*_Ro_ is the outdoor radon concentration; *E*_Rn_ is exhalation rate; $\lambda_{{{\text{Rn}}}}$ is decay constant and *v* is ventilation rate and in steady state conditions the equation takes the form

5 $$C_{{{\text{Rn}}}} = \frac{{E_{{{\text{Rn}}}} + vC_{{{\text{Ro}}}} }}{{\lambda_{{{\text{Rn}}}} + v}}$$

In general, in the moderate conditions the typical ventilation rates rages from 0.1 to 1.5 h^−1^. The radon decay constant $\left( {\lambda_{{{\text{Rn}}}} = 7.6 \times 10^{ - 3} \;{\text{h}}^{ - 1} } \right)$ is much lower than average ventilation rates prevailing in the dwelling. Hence neglecting $\lambda_{{{\text{Rn}}}}$ in the above equation, it becomes

6 $$C_{{{\text{Rn}}}} = \frac{{E_{{{\text{Rn}}}} }}{v} + C_{{{\text{Ro}}}}$$

It is clear from Eq. (6), that increase in exhalation rates and decrease in ventilation rates results in the accumulation of radon during night hours and vice versa.

However, the thoron concentration was found to be varying scattered because a small change in the environmental conditions results in large variation of thoron levels (see Fig. 3). This can be attributed to its shorter half-life or smaller diffusion length. The smaller diffusion length and poor air circulation obstruct the up surging of thoron, which causes low concentration of indoor thoron during night hours. The increased activity concentration of thoron in the morning hours can be attributed to circulating air currents that support in increasing the diffusion length^24^.

Mathematically, the expression for indoor thoron concentration, similar to Eq. (5), under steady state condition is given by

7 $$C_{{{\text{Tn}}}} = \frac{{E_{{{\text{Tn}}}} + vC_{{{\text{To}}}} }}{{\lambda_{{{\text{Tn}}}} + v}}$$

where *C*_Tn_ is indoor thoron concentration; *C*_To_ is the outdoor thoron concentration; *E*_Tn_ is exhalation rate; *v* is ventilation rate and $\lambda_{{{\text{Tn}}}}$ is decay constant. The shorter half-life of thoron (56 s) gives rise to a decay constant of 45.4 h^−1^. Average ventilation rate existing in the dwelling is much smaller than $\lambda_{{{\text{Tn}}}}$ hence the Eq. (7) can be simplified to

8 $$C_{{{\text{Tn}}}} = \frac{{E_{{{\text{Tn}}}} }}{{\lambda_{{{\text{Tn}}}} }}$$

According to Eq. (8), the indoor thoron concentration is independent of ventilation and the concentration should increase with increase in exhalation rates. However, the results obtained in the present investigation (Fig. 3) are contradicting this. Due to higher decay constant, thoron quickly decays to polonium before the homogeneous mixing of the gas. The lesser diffusion length the thoron leads concentration of gas to decrease rapidly with the distance from source (floor, wall or roof in this case). Assuming the surfaces as the source of thoron, the concentration of thoron as a function of distance from source is given by Doi et al.^25^.

9 $$C_{{{\text{Tn}}}} \left( x \right) = C_{{{\text{Tn}}}} \left( 0 \right)e^{{\left( { - \frac{x}{L}} \right)}}$$

where $C_{{{\text{Tn}}}} \left( x \right)$ is concentration of thoron at “*x*” distance from source; $C_{{{\text{Tn}}}} \left( 0 \right)$ is concentration of thoron at source and $L = \left( {D/\lambda_{{{\text{Tn}}}} } \right)^{1/2}$ is diffusion length; *D* is diffusion coefficient in air. With the given constant values for *D* and $\lambda_{{{\text{Tn}}}}$; the concentration of thoron at 1 m from the source should decrease to less than one percent of the initial concentration^23^. However, studies showed that the decrement is not that rapid and thoron concentration observed to drop to 10–40% at 1 m distance from the source^24–26^. The air circulation in the room promotes the thoron gas to diffuse more distance. Thus, during the night air circulation is lower and hence the concentration of the gas suppressed and it increases with increase in air currents during dawn/dusk. In summary, concentration of radon increases with decrease in ventilation rates whereas the thoron gas concentration is aided with air exchange rates in the dwellings. This property discriminates both the isotopes and can be used in separating them.

### Seasonal variation

**Figure 4:**
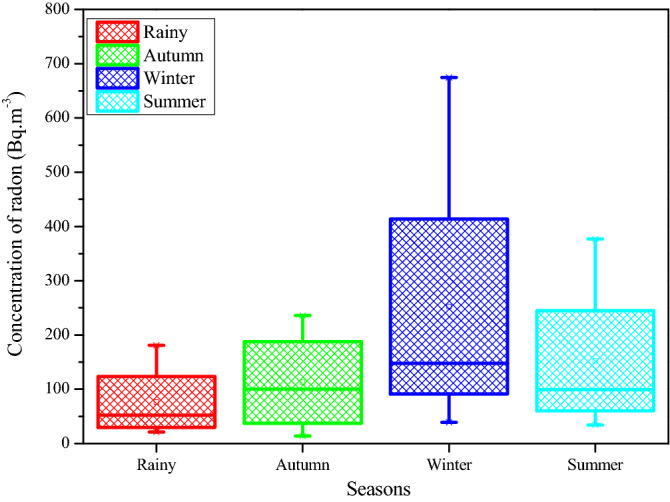
Seasonal variation of ^222^Rn levels in the study area.

**Figure 5:**
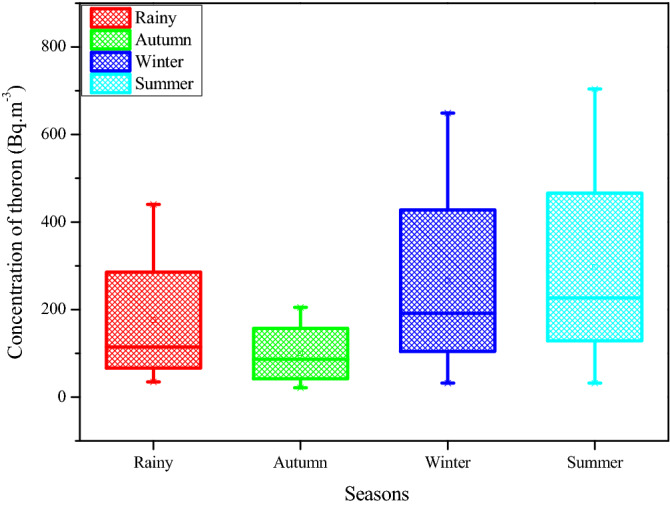
Seasonal variation of ^220^Rn levels in the study area.

The seasonal variation of residential radon/thoron was monitored by time integrated technique. The dosimeters were installed in the dwellings covering the four seasons of the year and estimated seasonal variation of average radon and thoron concentration levels for the dwellings is shown in Figs. 4 and 5. The radon levels were found to be higher in the winter season than the other seasons. This is because most of the dwellers in the winter usually close all the windows to conserve the energy and as a consequence the radon gas concentration turned out to be potential in the dwellings. The ratio of radon levels between winter and summer season is found to be 1.36 and this is similar with the earlier findings^27–29^. Rationales for elevated radon levels in winter can be attributed mainly to (1) The life style of the people in the forest environment that limits the air circulation inside the dwellings in winter season by closing the ventilation systems longer time compared to other seasons (2) the enhanced emanation rate of radon from floor and walls because of the positive temperature gradient exists between inside and outside the dwellings. The variation of thoron levels with seasons in certain cases is unlike the radon because of its unusual behavior results an inversion current flow of the thoron gas than other conventional gases, may be attributed to its short half-life^30,31^. This might be the reason the ratio of measured thoron levels between the winter and summer is found to be little lower than the unity (0.92).

### Variation of activity concentrations with roof, floor and wall of the dwellings

**Figure 6:**
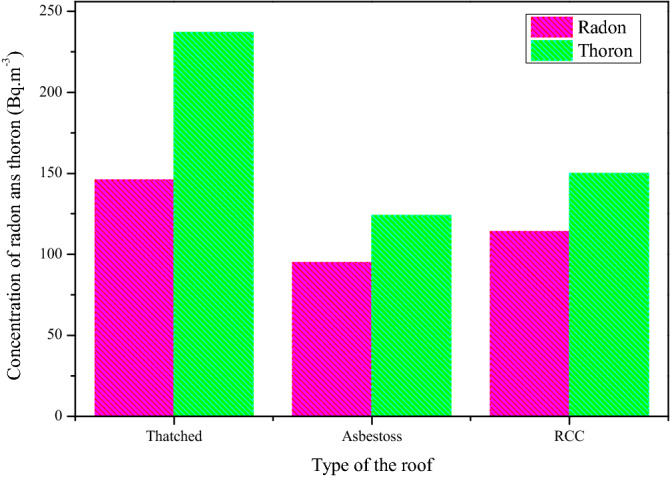
Variation of indoor radon/thoron concentration with different types of materials used for roof.

It is recognized that the airborne radioactivity levels, are mainly because of not only the soil underneath of the dwelling, but also noticeably depends upon the materials used for the construction and ventilation parameters. To understand this, an attempt is made to estimate the radon and thoron levels in different types of dwellings with respect to the roof, floor and walls. The variation of radon and thoron levels in different type of roofs is shown in Fig. 6. It is found the levels are high in Rein Forced Concrete (RCC) and thatched roof houses when compare to asbestos houses. This is can be expected due to the ventilation system in the asbestos houses usually at lesser height from the roof results well ventilation, as a consequence the dispersion of air taking place faster than the other type of houses and moreover in the present study it is observed that the size of these houses considerably small and most of this type of dwellings are single rooms. The higher radiation levels in RCC and thatched roof houses is attributed to poor ventilation since (1) RCC houses are comparatively large in size and generally have one window results poor ventilation (2) Thatched houses usually have no windows and the access is also narrow in size probably results very poor ventilation. Further the dwellings are also analyzed based on the floor and walls of the dwellings and the measured average radon and thoron levels are given in Table 2. It is inference that the radon and thoron levels are found to be higher in the dwellings constructed either floor or walls with mud and this findings are consistent with the earlier results^6,32,33^. The difference of the thoron levels between the mud and cement floor, mud and cement wall are relatively higher than radon levels of identical floor and wall, this may be due to the concealing effect of thoron emanation by the cement because of its shot half-life.

**Table 2:** The activity concentration of radon (^222^Rn) and thoron (^220^Rn) (Bq m^−3^).

	Radon				Thoron			
	Floor		Wall		Floor		Wall	
	Mud	Cement	Mud	Cement	Mud	Cement	Mud	Cement
Min	22	14	22	14	44	28	44	21
Max	675	377	675	377	667	704	667	704
Avg	122	118	124	117	196	164	207	151
SD	107	78	54	77	155	143	181	146

### Inhalation dose

In the calculation of annual effective inhalation dose due to radon and thoron gases, the important parameters are equilibrium and occupancy factors. The significant parameter used in the estimation of dose is the equilibrium factor (EF) related with ventilation rate, which is measure of disequilibrium between radon and its daughters; thoron and its daughters. In the present investigation the average EF for inhalation dose is used as 0.40 for radon^1^ and for thoron is 0.12^12^. Occupancy factor is another principal factor in the estimation of inhalation dose. United Nations Scientific Committee on Effects of Atomic Radiation^1^ suggested the occupancy factor of 0.8 (7000 h spent indoors in a year, which is opting for professionals or occupational workers). However, in rural areas the dwellers usually leave the house in early hours to the fields and will be back only in the evening hours. Therefore, the present study conducted in a forest village, it is appropriate to consider an occupancy factor of 0.6 (5250 h spent indoors in a year, which is opting for self-employed, agriculture labourers, farmers). Table 3 presents the inhalation dose received by the habitats in the study area calculated with the occupancy factor of 0.6. The table also presents the dose values computed with the occupancy factor of 0.8 for comparison purpose. The estimated inhalation dose due to radon in the study area varies from 0.26 to 12.77 mSv y^−1^ with a geometric mean of 1.78 mSv y^−1^ and due to thoron it varies from 0.53 to 17.76 with the geometric mean of 3.05 mSv y^−1^. The estimated dose is higher when compare with the global average inhalation dose due to radon and thoron^1^.

**Table 3:** Annual effective dose due to inhalation of radon and thoron (mSv y^−1^) with occupancy factors (OF) = 0.6 and 0.8.

Parameter	OF = 0.6		OF = 0.8	
	^222^Rn	^220^Rn	^222^Rn	^220^Rn
Geometric mean	1.78	3.05	2.37	4.07
Minimum	0.26	0.53	0.35	0.71
Maximum	12.77	17.76	17.03	23.68
Mean	2.27	4.29	3.03	5.72
Standard deviation	1.87	4.01	2.50	5.35

## Conclusions

The calculated geometric mean values of activity concentration of radon and thoron are found to be 94 Bq m^−3^ (arithmetic mean122 ± 98 Bq m^−3^) and 121 Bq m^−3^ (arithematic mean166 ± 159 Bq m^−3^) in the present study area and are observed to be higher than global average concentration of 40 Bq/m^−3^ and 10 Bq/m^−3^, respectively. The distribution plots of radon and thoron levels show an asymmetrical (lognormal) around the average activity. The diurnal studies indicate the radon levels are high during the day and night time while the same downturn during dawn and dusk. Contrary to the radon the variation of thoron activity is explained on the basis of air circulation rates. Among the different types of dwellings, the airborne radiation levels were found to be relatively high in dwellings constructed with mud compared to other types. The annual average inhalation dose exposure due to radon and thoron to community in the investigated area is found to be relatively higher.

## Data Availability

The datasets used and/or analyzed during the current study are available from the corresponding author with request.
